# Does A Flavoured Extra Virgin Olive Oil Have Higher Antioxidant Properties?

**DOI:** 10.3390/antiox11030550

**Published:** 2022-03-14

**Authors:** Enrique Jacobo Díaz-Montaña, María Barbero-López, Ramón Aparicio-Ruiz, María T. Morales

**Affiliations:** Department of Analytical Chemistry, Faculty of Pharmacy, University of Seville, Calle Profesor García González, 41012 Seville, Spain; edmontana@us.es (E.J.D.-M.); marbarlop6@alum.us.es (M.B.-L.); aparicioruiz@us.es (R.A.-R.)

**Keywords:** virgin olive oil, phenols, flavoured oils, rosemary, basil, oxidation, storage

## Abstract

Extra virgin olive oil is highly appreciated worldwide for its healthy and organoleptic properties. From the variety of compounds present in the oil, phenols stand out, not only for producing the bitter-pungent perception but also for their antioxidant properties, which contribute to human health protection. The addition of plants can change the phenolic profile due to a migration of plant antioxidants to the oil. The aim of this work was to study the evolution of the oxidative process of extra virgin olive oil under mild storage conditions for 8 months, monitoring the individual content of 15 phenols by High Performance Liquid Chromatography (HPLC) and the changes of the phenolic profile of the non-flavoured oil compared with the same flavoured (rosemary and basil) oil. The oxidative alteration was more marked in virgin than in flavoured oils, where it happened slowly. Throughout storage, the behaviour of the phenols varied, resulting in a decrease in their concentration, except in the case of tyrosol and hydroxytyrosol. The addition of plants had an antioxidant effect, slowing down the oxidative process, which prolongs the shelf life of the flavoured oil compared to the unflavoured oil. Furthermore, multivariate statistical analyses allowed the classification and differentiation of the different samples.

## 1. Introduction

The Mediterranean diet is a healthy and nutritional dietary pattern in which olive oil plays a main role in the reduction of all-cause mortality but mainly the risk of diseases related to oxidative damage, such as coronary heart disease, diabetes, cell aging, and some types of cancer [1]. This damage is avoided thanks to the combination of phenols, tocopherols and other minor compounds, as well as the proportion between monounsaturated (oleic acid) and polyunsaturated fatty acids [2]. The most remarkable healthy property of the phenolic compounds is that they act as antioxidant agents, eliminating oxygen reactive species (free radicals). They can carry out this activity in various ways, forming chelates with the metal ions that participate in oxidative systems or breaking the free-radical chain reaction [3].

On the other hand, extra virgin olive oil (EVOO) organoleptic properties are largely responsible for its outstanding position in world gastronomy [4]. Its particular and highly appreciated flavour is due to a mixture of volatile and phenolic compounds that confer EVOO a pleasant aroma and a delicious taste [5,6]. Volatile compounds contribute to aroma, while phenols are responsible for taste, mainly the perceptions of bitterness and pungency [5,7].

Phenolic compounds constitute a wide and varied group of molecules that have an aromatic ring attached to one or more hydroxyl groups. A large variety of these structures, which are split into hydrophilic or lipophilic, has been determined in EVOO. The hydrophilic group is constituted by phenolic acids, simple phenols, oleuropein and ligstroside derivatives, flavonoids, lignans and hydroxy-isochromones [8], several of which are also present in other vegetables and fruits, with the exception of oleuropein derivatives that are only found in Oleaceae family [9]. The phenolic composition differs among EVOOs due to the olive variety, the olive ripeness when harvesting, rainfall in autumn, altitude of cultivars and, mostly, the time of harvesting and the storage conditions [10]. It is so because the shelf life of any lipid is closely related to an oxidative process—accelerated or slowed down by its storage conditions—and phenols are the first defence line against oxidation, followed by non-saturated fatty acids [11].

Consequently, during EVOO storage, the phenolic profile changes due to the oxidation process [12], and the total initial concentration can diminish up to 73% [13], depending on the time and condition of the storage. The studies about the deterioration of phenols in olive oils, however, should be carried out under storage conditions close to the commercial routine because their conclusions usually differ from those performed under accelerated storage conditions [14]. On the other hand, the phenolic profile can undergo significant changes due to the addition of numerous plants and herbs to EVOOs [15,16], which is a tradition of the Mediterranean areas, that modify not only the phenolic profile but also their sensory and healthy properties [17].

Most of the works are focused on how to dress olive oils and their shelf life, but only a few ones study the quality parameters for the virgin olive oil categories [18] and even less papers are centred on the evolution of the main individual phenolic compounds for a long period of time.

Therefore, the objective of this work was to compare the evolution of 15 phenolic compounds in non-flavoured and flavoured EVOOs for 8 months and to observe the antioxidant effect of the addition of basil (*Ocimum basilicum* L.) and rosemary (*Rosmarinus officinalis* L.) by slowing down the ineluctable oxidation process.

## 2. Materials and Methods

### 2.1. Samples

An EVOO (var. Manzanilla) from the 2020/2021 season was purchased from the local market (Seville, Andalusia, Spain). The oil was filtered and packed into 100 mL transparent glass containers. After the measurement of the initial virgin olive oil sample (VOO-0), which was the only one without storage, the samples were prepared and stored, finally obtaining 24 samples, divided into 3 groups: (i) 8 samples containing only EVOO (VOO), (ii) 8 samples containing EVOO dressed with dried rosemary (ROO, Rosemary Olive Oil) leaves at 5% *w*/*w* (iii) and another set of 8 samples containing EVOO dressed with dried basil (BOO, Basil Olive Oil) leaves at 5% *w*/*w*.

The leaves of the aromatic plants were collected from the countryside of Seville (Spain), and they were oven-dried at 60 °C for 10 h [19].

The storage of the 24 samples for 8 months was under moderate conditions, emulating the storage in a supermarket. The temperature was in the range of 15.7 ± 3.6 ºC and the light intensity at 400–600 lx. The samples were exposed to light/darkness cycles of 12 h. Every month a bottle of each group (VOO, ROO and BOO) was analysed by duplicate, and then discarded. The analysis included 15 individual phenols and the physical-chemical quality parameters.

### 2.2. Quality Parameters

Free acidity (FA), peroxide value (PV) and specific extinction values (K_232_, K_270_ and ∆K) were determined according to European Union standard methods [18].

All the reagents used in this study were of pure analytical grade. Methanol, hexane and ethyl acetate were purchased from Panreac (Barcelona, Catalonia, Spain) and p-hydrophenylacetic acid, o-coumaric acid, orthophosphoric acid and acetonitrile purchased from Merck (Darmstadt, DE, Germany).

### 2.3. Determination of Phenol Compounds

The analysis and determination of the phenolic compounds was carried out using a previously developed analytical methodology [20], later modified and validated [7].

A standard solution (0.5 mL) of p-hydroxyphenylacetic (0.12 mg/mL) and o-coumaric acid (0.01 mg/mL) in methanol was added to the filtered samples (2.5 g). A rotary evaporator (rotvap) at 30–35 °C under vacuum was used to evaporate the solvent and the oily residue was dissolved in 6 mL hexane.

The diol-bonded phase cartridge was conditioned according to Mateos et al. [20]. After sample loading, the phenols retained in the cartridge were eluted with 10 mL methanol, and the solvent was evaporated in a rotvap at 30–35 °C. Finally, the residue was dissolved in 500 µL methanol–water (1:1 *v*/*v*) and a filtered aliquot (20 µL) of the final colourless solution was injected into the HPLC system (LaChrom Elite, Tokio, Japan), equipped with a diode array detector. The column was a Lichrospher 100 RP-18 (4.0 mm i.d. x 250 mm; 5 µm, particle size) (Darmstadt, DE, Germany) maintained at 30 °C. The gradient elution, at a flow rate of 1.0 mL/min, was achieved by using the following mobile phases: a mixture of water/ortho-phosphoric acid (99.5:0.5 *v*/*v*) (solvent A) and methanol–acetonitrile (50:50 *v*/*v*) (solvent B). The change in solvent gradient was programmed as follows: from 95% (A)–5% (B) to 70% (A)–30% (B) in 25 min; 62% (A)–38% (B) in 15 min; 55% (A)–45% (B) in 5 min; 47.5% (A)–52.5% (B) in 5 min and 100% (B) in 5 min, followed by 5 min of maintenance. The chromatographic signals were acquired at 235, 280, and 335 nm.

The quantification of the phenols, cinnamic acid, and lignans was carried out at 235 and 280 nm, using p-hydroxyphenylacetic acid as internal standard (IS 1). The quantification of flavones was conducted at 335 nm by using o-coumaric acid as internal standard (IS 2). The determination of the 15 phenols was performed using the response factors from Mateos et al. [20].

### 2.4. Statistical Analysis

The whole set of data were imported to Excel 2016 from the instrument, and STATISTICA v.8 package (StatSoft, Tulsa, OK, USA) was used to carry out the data treatment and the statistical analysis. Brown–Forsythe test was used, and it allowed to select compounds that showed significant differences in their relative concentration (*p* < 0.05) among the groups.

Cluster analysis of the control samples (VOO) characterized by 15 phenols was performed by a complete standardization of the data and was implemented with the City-Block (Manhattan) distance measure and the single linkage amalgamation rule.

In addition, a principal component analysis (PCA) was carried out as unsupervised procedure to explore the result and determine the discrimination power. Firstly, we applied PCA to all the variables, and removed those with very low correlation with the first two factors as it can be supposed that they do not contribute to distinguish the whole set of samples in any manner.

## 3. Results and Discussion

For years, it was well known that some plants and herbs had antioxidant properties because of their amount of certain phenolic compounds that decreased the formation of hydroperoxides, which is related to auto-oxidation processes [21]. Among the large set of thousands of different olives, there are cultivars with low concentration of phenols. These olives are usually consumed as table olives but, from time to time, they are milled to produce EVOOs, which have shorter shelf life. Those virgin olive oils are usually potential candidates to be dressed with herbs or plants, which allows to expedite the implementation of Mediterranean recipes as well as to mask any early sensory defect perception [22]. Thus, EVOO var. Manzanilla [23] was selected for three basic reasons: (i) it has always been a good candidate to be flavoured, particularly in years with high production of table olives; (ii) it is usually sold as unfiltered EVOO (also named veiled or cloud EVOO), and it is a perishable colloidal system since it quickly worsens the initial quality due to the hydrolysis of triacylglycerols, the oxidation of phenols and the off-flavour-formation [24]; and (iii) its shelf life is hence shorter than other mono-varietal olive oils, which allowed reducing the experiment by months without losing scientific support.

### 3.1. Quality Parameters

Quality criteria of virgin olive oil categories are well described in international and national regulations that are based on the Trade Standards of the International Olive Council [25]. The three most widely applied quality criteria are free acidity, peroxide value, and absorbance in ultra-violet, and they are used to control the natural evolution of any EVOO to VOO with time from olive milling. Since the study aimed to evaluate changes of the phenolic composition in the set of oils (VOO, ROO, BOO) throughout storage, those chemical indexes were determined in the control sample prior to carrying out the experiments, and then each 30 days.

According to the results, the initial sample (VOO-0) was initially classified as EVOO because the values of the indexes were below their limits [25]. Peroxide values also remained below the limit, 20 meq O_2_/Kg, in the whole experiment for all the samples, in either flavoured or unflavoured oils. Concerning the absorbance in ultra-violet the values of K_232_ were also below the limit throughout the whole experiment, but it was not the case for the values of K_270_. Thus, the sample flavoured with rosemary exceeded the limit from the third month onwards, which is not an abnormal result, as K_270_ of EVOOs—with high content of phenols—exceeded the International Olive Oil Council (IOC) limits when stored in green glass for more than 5 months [26].

**Figure 1:**
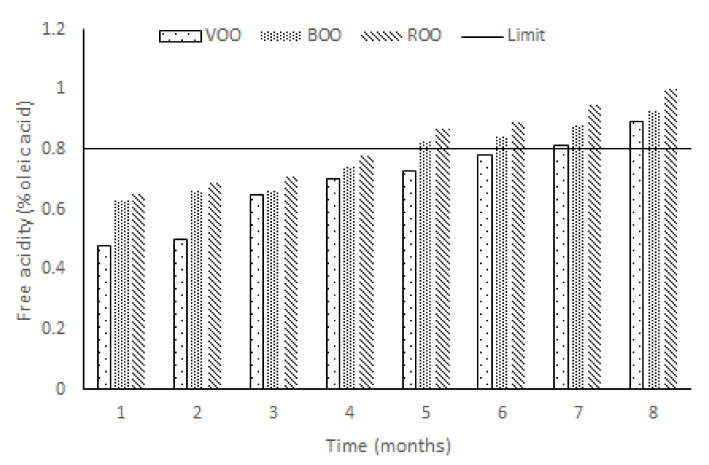
Bar graph for free acidity (FA) of the three samples—virgin olive oil (VOO), virgin olive oil flavoured with basil (BOO), and virgin olive oil flavoured with rosemary (ROO)—during the storage experiment of eight months. The limit of FA for extra virgin olive oils (EVOO) is 0.8% [25].

Free acidity (FA) is by far the most implemented criteria for qualifying virgin olive oil categories. Figure 1 shows that FA increased during the storage of the three sets of samples (VOO, ROO, BOO) in a linear trend from below to above 0.8, which is the maximum value for EVOOs. This may come as a surprise in filtered EVOOs, but it is less strange in veiled EVOO var. Manzanilla because of the progressive hydrolysis of the triacylglicerides in unfiltered oils [24]. Thus, the control samples (VOO) reached a value above 0.8 in the seventh month, when this usually may occur in commercial EVOOs after the “best before” date, around one and half or two years after bottling. Fortunately, it does not automatically imply any increase in the oxidation process of the oil or a decrease of its total phenol content.

Flavoured samples (ROO, BOO) have a supplementary amount of water that facilitates an increment of the hydrolytic processes [19], generating free fatty acids, mainly responsible for free acidity, as is widely known. In addition, the effect on the free acidity of the migration of organic acids (such as rosmarinic and carnosic acids) from the vegetal material to olive oil is much lower than by free fatty acids (FFA) coming from the triacylglycerols hydrolysis [27]. Thus, flavoured EVOOs (ROO, BOO) showed a higher FA in comparison with control samples, which is attributed to the presence of vegetal material in the oil, because even though were dried, they still have a small percentage of water. In this sense, olive oil dressed with a plant (rosemary) always showed higher FA than olive oil dressed with an herb (basil) along the whole process.

### 3.2. Phenolic Compounds

The quantification of phenols was conducted with two internal standards (Table 1), of which basic analytical quality parameters related to precision (repeatability and intermediate precision) were determined prior to their use in the planned work. Thus, the analysis of internal standards was repeated five times—within a single work session—for the repeatability evaluation. The intermediate precision was evaluated by the analysis in different work sessions in nine non-consecutive days. As shown in Table 1, relative standard deviation (RSD) (%) were below 6% in both repeatability and intermediate precision, which are in line with other authors [20,28].

On the other hand, Table 2 shows the phenol names, their retention times (t_R_), and the concentrations (mg/Kg) of the non-stored sample (VOO-0). These phenols are clustered as simple phenolic acids (vanillic, p-coumaric and cinnamic acids), flavonoids (apigenin and luteolin), simple phenols (hydroxytyrosol, hydroxytyrosol acetate, tyrosol, tyrosol acetate, and vanillin), lignans (pinoresinol), aldehydic and dialdehydic forms of oleuropein and ligstroside (3,4-DHPEA-EA, p-HPEA-EA, 3,4-DHPEA-EDA and p-HPEA-EDA), and oleuropein and ligstroside aglycone. Concentration values are inside the ranges determined in other monovarietal EVOOs [7] when quantified with the same or similar analytical method.

### 3.3. Changes of Phenolic Profile during the Storage Experiment

Tens of papers have discussed that the phenolic profiles of VOOs vary during their shelf life, whichever storage system or cultivar, having individual compounds increase or decrease in their concentrations [10,13,14]. According to Esposto et al. [26], the sums of the concentrations of the oleuropein derivatives (e.g., 3,4-DHPEA, 3,4-DHPEA-EA, 3,4-DHPEA-EDA) diminish with the storage time, and, on the contrary, the sum of the concentration of ligstroside derivatives (e.g., p-HPEA, p-HPEA-EDA) does not seem to vary too much. However, our study is the first to describe individual changes in the secoiridoid derivative groups during the shelf life of flavoured EVOOs.

**Figure 2:**
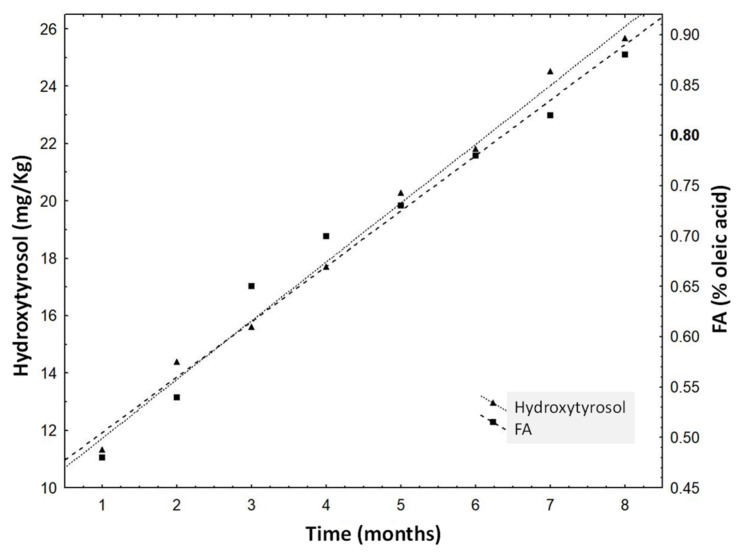
Free acidity (FA) of the control samples throughout the storage experiment. The limit of FA is 0.8% for EVOO [25].

The four oleuropein-derivate compounds changed significantly (*p* < 0.05) in the control samples (VOO) during the experiment: hydroxytyrosol and 3,4-DHPEA-EA increased their concentrations while the concentration of 3,4-DHPEA-EDA and hydroxytyrosol acetate diminished. Hydroxytyrosol is the last derivative that is generated in the oxidation and hydrolysis processes of the oleuropein-derivates, and many works have reported that its concentration increases in oxidation processes [29]. Figure 2 shows that hydroxytyrosol concentration increased linearly (R^2^ = 0.97) in the storage experiment of VOOs. The concentrations of hydroxytyrosol and the FA were well correlated (R^2^ = 0.93), as can been deduced from Figure 2. This correlation is a consequence of the fact that free fatty acids, and partially hydroxytyrosol, result from hydrolysis.

Table 3 shows the evolution of the concentrations of oleuropein and ligstroside derivatives in the VOO samples in four particular months (first, fourth, fifth and eighth) of the whole storage process; selection of these months was not arbitrary but based on statistical results later explained. They are tyrosol (p-HPEA), p-HPEA-EA, p-HPEA-EDA and tyrosol acetate, as ligstroside-derived compounds, and hydroxytyrosol (3,4-DHPEA), 3,4-DHPEA-EA, 3,4-DHPEA-EDA and hydroxytyrosol acetate as oleuropein-derived compounds. According to Migliorini et al. [29], ligstroside- and oleuropein-derived compounds are thought to be subjected to degradation, resulting in hydrolytic and oxidative changes of both enzymatic and non-enzymatic nature. The named series of phenols do not show similar behaviours throughout the storage process, with the exception of p-HPEA and 3,4-DHPEA, the concentrations of which increased during the storage time, and the dialdehydic forms (p-HPEA-EDA and 3,4-DHPEA-EDA), the concentrations of which diminished (Table 3). A significant increase (*p* < 0.05) of tyrosol was observed from the fifth month, while the increase of the concentration of hydroxytyrosol was observed from the fourth month. Four of the compounds (Table 3) showed significant differences (*p* < 0.05) between the two central months, and the eighth month presented a significant difference (*p* < 0.05) in concentrations between the initial and the final month. In contrast to Esposto et al. [26], the sum of ligstrosides (tyrosol and p-HPEA-EDA) showed a significant decrease (*p* < 0.05) after eight months of storage.

**Figure 3:**
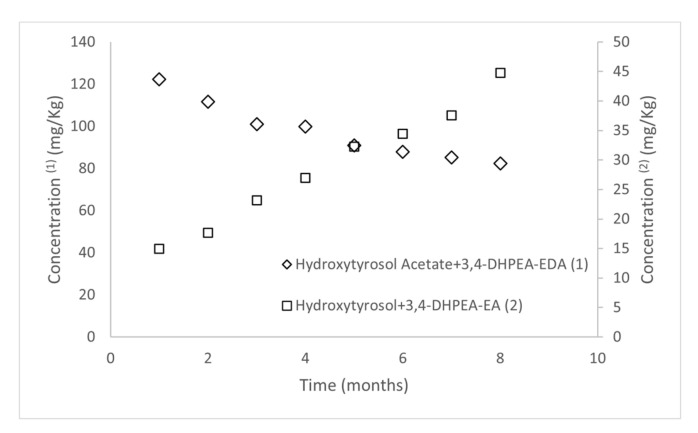
Evolution of the sum of oleuropein-derivate which concentrations increase (2) (3,4-DHPEA-EA and hydroxytyrosol), and the sum of oleuropein-derivate which concentrations decrease (1) (3,4-DHPEA-EDA and hydroxytyrosol acetate) throughout storage.

With respect to the profiles of those derivatives from oleuropein and ligstroside throughout storage, the concentrations of 3,4-DHPEA-EDA diminished in a linear manner up to 32.83% from the initial value. Conversely, the increase of the concentrations of hydroxytyrosol was partially possible because the dialdehyde degrades to it [13]. Additionally, there was a good relationship between the sum of the concentrations of hydroxytyrosol acetate and 3,4-DHPEA-EDA during the eight months of the experiment, as well as for the sum of the concentration of hydroxytyrosol and 3,4-DHPE-EA with the storage time. However, the relation between the two sums is inversely proportional, due to the degradation of 3,4-DHPEA-EDA and hydroxytyrosol acetate to hydroxytyrosol and 3,4-DHPEA-EA, as displayed in Figure 3.

In addition to oleuropein and ligstroside derivatives, another phenol that showed significant differences (*p* < 0.05) in long storage was cinnamic acid. Cinnamic acid has also been studied in other edible oils (e.g., palm oil) because it is one of the few phenols that can be metabolized by fungi (e.g., *Phomopsis liquidambari*) [30]. Cinnamic acid concentration decreased in VOO (64.67%), as it degrades to its hydroxylated derivatives, such as ferulic acid, caffeic acid and p-coumaric acid. This degradation correlated perfectly (R^2^ = 0.93) with the significant increase (*p* < 0.05) of p-coumaric acid (64.17%).

**Figure 4:**
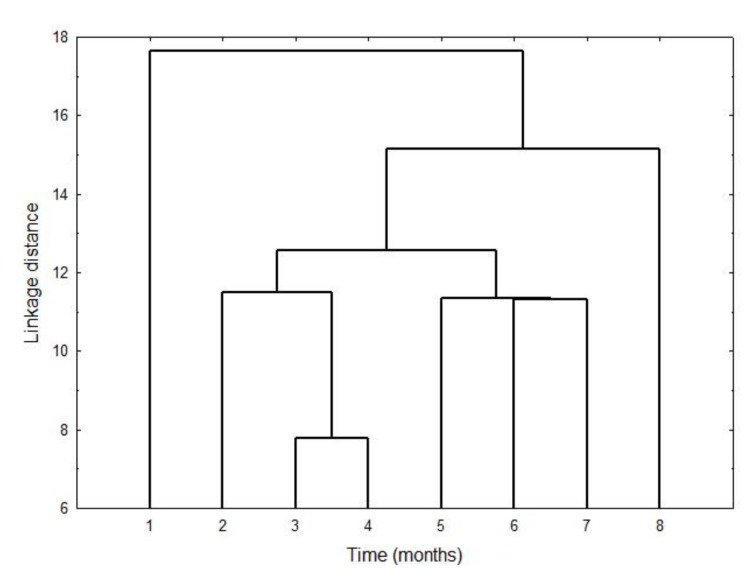
Cluster analysis of the VOO samples characterized by 15 phenols. Cluster of the full standardized data was implemented with City-Block (Manhattan) distance measure and the single linkage amalgamation rule.

Figure 4 displays the result of applying a cluster analysis to control samples of the experiment, characterized with the full set of 15 phenols. The result is a chronological classification of the phenolic profiles determined throughout storage, the initial profile being quite different from all the rest. In fact, the phenolic profiles of the first and the last months were different from the two basic groups, in which the rest of the profiles clustered chronologically. Profiles of the fifth, sixth and seventh months were different but with some common information, as can be deduced from Figure 4. They were different from the third and fourth months, which were similar to each other, and different from the profile corresponding to the second month.

Cluster analysis showed again that phenolic compounds contain information directly related to the degree of alteration of the samples, and that this alteration occurs gradually [10]. All this indicates that the phenolic compounds may be suitable markers of the oxidative stability of VOO.

Once we described the results of the control samples, the next step was their comparison with flavoured samples (BOO, ROO) throughout storage, the matrix of which was also the initial control sample. Thus, for the analysis of the influence of storage time on the initial phenolic concentration of BOO and ROO samples, the phenols were clustered into three great groups: (i) oleuropein and ligstroside derivatives; (ii) cinnamic acid, luteolin, pinoresinol and p-coumaric acid; and (iii) apigenin, vanillic acid and vanillin. Table 4 displays the mean (± SD) concentrations of the oleuropein and ligstroside derivatives of phenols in the flavoured samples in four selected months of the storage as was shown with VOO samples; the selection is explained in Figure 4.

Concerning oleuropein and ligstroside derivatives (Table 4), flavoured olive oil (FOO) samples presented a significant decrease (*p* < 0.05) in the concentrations of 3,4-DHPEA-EDA and hydroxytyrosol acetate. The degradation of complex compounds, although significant (*p* < 0.05), occurred more slowly, which caused the concentration of hydroxytyrosol, the final degradation compound, to not increase significantly (*p* < 0.05).

As displayed in Table 4, the same trend can be observed between the VOO and FOO both in oleuropein derivatives on the one hand, and in those derived from ligstroside on the other. However, unlike VOO, FOO did not show a significant increase (*p* < 0.05) in hydroxytyrosol, and this may be due to the fact that in flavoured samples the other compounds oxidize slower, making the last oleuropein derivative (hydroxytyrosol) not vary significantly (*p* < 0.05).

On the other hand, tyrosol increased in VOO, BOO and ROO, but only in BOO this increase was not significant (*p* < 0.05), which, based on the logic of hydroxytyrosol, would mean that basil is capable of slowing down the oxidative process more than rosemary, and therefore tyrosol did not increase significantly (*p* < 0.05).

The second group, despite varying slightly, did not show significant differences (*p* < 0.05) during storage in any type of sample, even though they were considered for statistical analysis to see how they affected the classification of the samples. In addition, these compounds have an impact in sensory perception and can be used also as a fingerprint of VOO, so they should never be omitted [31].

The other compounds made it possible to differentiate VOO samples from flavoured samples, enhancing their differences. Cinnamic acid presented significant differences (*p* < 0.05) between the initial and final concentration for the three types of samples. A decrease in the concentration was observed, due to degradation to oxidation compounds, of 64.7%, 63.5% and 61.7%, for VOO, BOO and ROO, respectively. In addition, it was observed that for the samples without flavouring the slope was greater, which indicates that the degradation of these compounds was faster, which is justified by the protective role of the antioxidant compounds present in plants. In the case of flavoured samples, the relationship between cinnamic acid and p-coumaric acid was not observed, due to the migration of these compounds from the plants to the oil.

**Figure 5:**
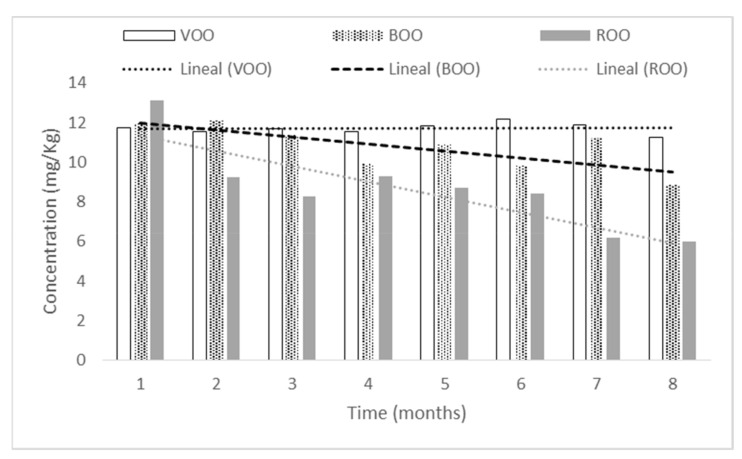
Luteolin concentration (mg/kg) in the storage process for the three samples.

Luteolin did not present significant differences (*p* < 0.05) throughout storage, but there was a decreasing trend in flavoured oils, while in VOO luteolin remained with little variation (Figure 5). Although luteolin is present in both species (basil and rosemary) and migration to oil occurs, luteolin can suffer a wide range of transformations (hydroxylation, methyloxydation, hydrogenation, methylation, among others) due to different enzymatic activities present in both species, therefore flavoured samples showed a decrease in their concentration [32,33].

Pinoresinol suffered a slight downwards variation in concentration (10.7%) for the VOO sample, which agrees with Esposto et al. [34], while there was a significant increase (*p* < 0.05) in the flavoured samples. This increase can only be due to the migration of this compound from the plants to the oil. It is found in leaves and especially in branches (woody part), so it is logical to observe that rosemary, which has a bigger woody part, presents a higher increase in its concentration [35,36].

### 3.4. Multivariate Statistical Analysis

**Figure 6:**
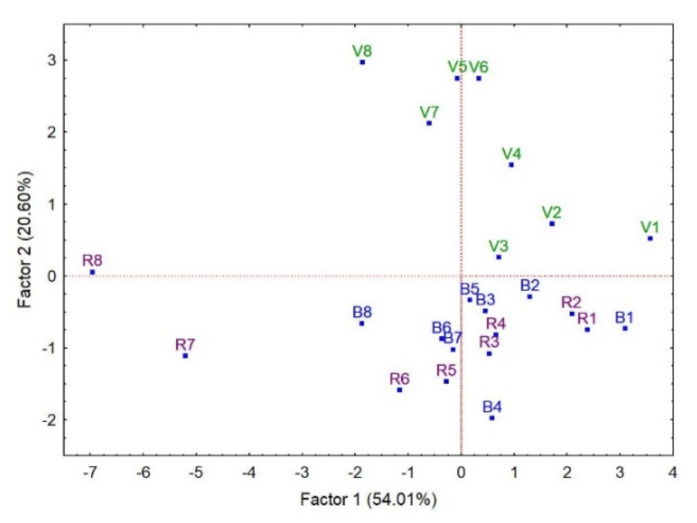
Principal components analysis of control virgin olive oils (V1-V8) and flavoured with basil (B1-B8) and rosemary (R1-R8). Numbers mean the month order in the storage experiment. Data were fully standardized to diminish the weight of the phenols with high vs. with low concentrations in the statistical analysis.

Multivariate analysis helps to understand the behaviour of several variables as a set instead of individually. However, not all the variables usually contribute to the objective of the study, and a filtering system has to be applied to select those with interesting information from the others that only contribute noise. There are several univariate algorithms that can determine the variables that are statistically significant for the work’s objective, e.g., Brown–Forsythe. However, the multivariate procedure of principal component analysis (PCA) was applied because it is an unsupervised procedure. Thus, firstly, a PCA was applied to all the variables, and those with very low correlation with the first two factors were removed, as it can be assumed that they do not contribute to distinguish the whole set of samples in any manner. Those removed were tyrosol acetate, p-HPEA-EDA, p-coumaric and vanillic acids, and apigenin. Next, PCA was applied to the rest of variables, with the result that Figure 6 displays.

At first sight, Figure 6 shows that Factor 1 is related with the time of storage (from right to left) while Factor 2 is able to distinguish flavoured vs. non-flavoured oils with the information from phenols. It is also remarkable that the samples of seven and eight months of the virgin oils flavoured with rosemary are quite different from the others in terms of the evolution of phenols during the storage experiment.

Hydroxytyrosol is the only phenol highly correlated with Factor 2 (R = 0.90). In fact, hydroxytyrosol concentration oscillated in the storage experiment between 11 and 26 mg/kg in VOO, between 11 and 14 mg/kg in BOO, and between 11 and 16 mg/kg in ROO. The first value corresponds to the first month and the second value to the last one of the process, which are, by the way, the minimum and maximum values of those sets of samples.

Factor 1, which is related with the time of storage, can be explained by five phenols. Three of them diminished their concentrations during the storage process (hydroxytyrosol acetate, 3,4 DHPEA-EDA and ligstroside aldehyde) and two phenols (oleuropein aldehyde and pinoresinol) increased their concentrations; all these phenols have a correlation of R = 0.9 with Factor 1, with the exception of hydroxytyrosol acetate with a correlation of R = 0.8.

The abrupt change in the concentrations of pinoresinol and ligstroside aldehyde in the samples ROO-7 and ROO-8 with respect to the previous six samples of ROO contribute to explain their behaviours as apparent outliers. In fact, the concentration of pinoresinol varied from 0.75 mg/kg, the mean of the first six samples, to 1.8 and 2.9 mg/kg of ROO-7 and ROO-8; the concentrations of ligstroside aldehyde changed from 5.7 mg/kg for the mean of the six previous months to 3.0 and 2.7 mg/kg for ROO-7 and ROO-8, respectively.

Factor 1 also shows a similitude between the order of the position of VOO samples in the PCA and in the cluster analysis of Figure 4. This sequence of samples is also observed in the samples of BOO and ROO, pointing out that there is a great similarity between the three groups, in particular at the beginning, probably because the migration of phenols from plant and herb had just started. Thus, the sector of Figure 6 that corresponds to the values of the X-axis (Factor 1) from 4 to 2 displays that the samples of the first month (VOO-1, BOO-1 and ROO-1) are clustered together. Next can be seen the samples of the second month, VOO-2, BOO-2 and ROO-2, though ROO-2 is in between samples from the first two months. Samples from the third and fourth month are in the sector constituted by the values 1 and 0 of Factor 1, leaving the negative values of Factor 1 to classify the samples of the sixth to eighth month, when there was an increase of the oxidation products.

## 4. Conclusions

The shelf life of edible oils is not easy to determine, even more so in virgin olive oil, due to the complexity of the matrix. Therefore, it is important to approach the studies with samples following the same process as in the usual storage methods, since different results can be obtained depending on the storage conditions. A mild storage condition for eight months, the most habitual among retailers and sellers, was enough to degrade the initial olive oil qualified as EVOO to a lower VOO quality. An increase of simple phenols was also observed at the expense of the degradation of more complex compounds, such as aglycones. Flavoured oils, however, showed a different behaviour, especially after the fourth month, due to the antioxidant effect exerted by the plants on the matrix, which resulted in a delay of its oxidative process. Unlike non-flavoured extra-virgin olive oil, the presence of plants (rosemary and basil) in flavoured oils resulted in a lower concentration of simple phenol compounds as a consequence of slowing the degradation of their precursors. Thus, individual phenols showed different evolutions throughout storage, and the differences in the contents of phenols of the non-flavoured samples were such that allowed to differentiate them in terms of their storage time by means of a cluster analysis. The phenolic content also allowed distinguishing between the flavoured and non-flavoured samples of the entire storage process by means of principal component analysis. Concerning flavoured samples, the addition of leaves of basil to EVOO seems to protect the oil against oxidation more than the addition of rosemary leaves.

These results shed more light on the already-investigated field of shelf life, in addition to generating more information to avoid possible fraud due to masking sensory defects. However, above all this study provides a new perspective on the importance that dressings and the plants used to flavour olive oils may have in prolonging and increasing the shelf life of edible oils.

## Figures and Tables

**Table 1:** Repeatability and intermediate precision of the internal standards (IS) used for the quantification pf phenols.

	Repeatability (RSD %)	Intermediate Precision (RSD %)
p-hydroxyphenylacetic acid (IS1)	1.95	3.38
o-coumaric acid (IS2)	2.15	5.93

**Table 2:** Phenols quantified in the initial control sample (VOO-0).

t_R_ (min)	Phenolic Compound	Concentration (mg/kg ± SD)
8.353	Hydroxytyrosol	10.79 ± 1.07
12.087	Tyrosol	13.68 ± 0.88
15.893	Vanillic acid	0.29 ± 0.03
17.660	Vanillin	0.01 ± 0.00
19.180	p-Coumaric acid	0.10 ± 0.04
22.213	Hydroxytyrosol acetate	3.49 ± 0.06
30.213	Dialdehydic decarboxymethyloleuropein aglycone (3,4-DHPEA-EDA)	118.18 ± 4.52
31.100	Tyrosol acetate	8.21 ± 0.50
35.980	Dialdehydic decarboxymethyligstroside aglycone (p-HPEA-EDA)	145.02 ± 7.31
36.787	Pinoresinol	0.93 ± 0.10
37.733	Cinnamic acid	0.14 ± 0.01
42.187	Luteolin	2.99 ± 0.99
43.920	Aldehydic decarboxymethyloleuropein aglycone (3,4-DHPEA-EA)	256.18 ± 8.52
47.507	Apigenine	3.54 ± 0.71
49.927	Aldehydic decarboxymethyligstroside aglycone (p-HPEA-EA)	135.01 ± 0.75

**Table 3:** Concentrations (mg/kg) of oleuropein and ligstroside derivatives (mean ± SD) of the control samples in four months of the storage process.

	VOO-1	VOO-4	VOO-5	VOO-8
Hydroxytyrosol	12.24 ± 1.57 ^a^	18.19 ± 0.71 ^b^	20.57 ± 0.42 ^b^	25.96 ± 0.71 ^c^
Hydroxytyrosol acetate	1.73 ± 0.10 ^a^	1.71 ± 0.03 ^a^	1.42 ± 0.01 ^b^	1.39 ± 0.03 ^b^
3,4-DHPEA-EA	2.72 ± 0.13 ^a^	8.77 ± 0.57 ^b^	11.69 ± 0.35 ^c^	18.83 ± 1.39 ^d^
3,4-DHPEA-EDA	120.57 ± 1.93 ^a^	98.17 ± 1.27 ^b^	89.52 ± 9.56 ^b^	80.98 ± 1.80 ^c^
Tyrosol	13.70 ± 0.60 ^a^	15.77 ± 0.62 ^a^	17.67 ± 0.27 ^b^	22.43 ± 0.87 ^c^
Tyrosol acetate	8.21 ± 1.15 ^a^	3.56 ± 0.38 ^b^	2.77 ± 0.18 ^c^	2.12 ± 0.01 ^d^
p-HPEA-EA	6.77 ± 0.26 ^a^	5.72 ± 0.49 ^a^	5.44 ± 0.98 ^a^	5.09 ± 0.04 ^b^
p-HPEA-EDA	143.23 ± 9.04 ^a^	104.23 ± 4.03 ^b^	99.34 ± 10.46 ^b^	96.72 ± 7.86 ^b^

Note: Values with the same letter (^a, b, c, d^) within the same row are not significantly different (*p* < 0.05). Experiments were set up in two repetitions and samples for each repetition were analysed in duplicate (*n* = 4).

**Table 4:** Concentrations (mg/kg) of the oleuropein- and ligstroside-derivates (mean ± SD) of flavoured samples (BOO, ROO) in four months of the storage process.

	BOO-1	BOO-4	BOO-5	BOO-8
Hydroxytyrosol	10.79 ± 1.40 ^a^	11.26 ± 0.22 ^a^	14.08 ± 0.81 ^a^	14.19 ± 0.17 ^a^
Hydroxytyrosol acetate	1.71 ± 0.01 ^a^	1.40 ± 0.03 ^b^	1.24 ± 0.03 ^c^	0.98 ± 0.11 ^c^
3,4-DHPEA-EA	2.67 ± 0.02 ^a^	6.29 ± 0.38 ^b^	7.93 ± 1.04 ^b^	12.54 ± 0.64 ^c^
3,4-DHPEA-EDA	108.85 ± 6.23 ^a^	77.70 ± 0.42 ^b^	70.65 ± 0.51 ^c^	67.57 ± 5.60 ^c^
Tyrosol	11.55 ± 0.91 ^a^	12.46 ± 0.35 ^a^	14.71 ± 1.15 ^a^	16.26 ± 1.64 ^a^
Tyrosol acetate	10.96 ± 0.67 ^a^	4.98 ± 0.91 ^b^	3.67 ± 0.18 ^b^	2.03 ± 0.01 ^c^
p-HPEA-EA	5.93 ± 0.02 ^a^	5.31 ± 0.92 ^a^	4.71 ± 0.57 ^a^	4.09 ± 0.34 ^b^
p-HPEA-EDA	119.96 ± 4.44 ^a^	89.38 ± 3.03 ^b^	76.24 ± 2.82 ^c^	72.53 ± 1.54 ^c^
	**ROO-1**	**ROO-4**	**ROO-5**	**ROO-8**
Hydroxytyrosol	10.38 ± 1.59 ^a^	12.23 ± 0.24 ^a^	12.90 ± 0.99 ^a^	15.37 ± 1.07 ^a^
Hydroxytyrosol acetate	1.51 ± 0.06 ^a^	1.16 ± 0.05 ^b^	1.10 ± 0.03 ^b^	0.83 ± 0.06 ^c^
3,4-DHPEA-EA	2.78 ± 0.15 ^a^	6.26 ± 0.07 ^b^	6.68 ± 0.74 ^b^	26.13 ± 1.55 ^c^
3,4-DHPEA-EDA	86.68 ± 7.57 ^a^	72.60 ± 2.22 ^a^	65.78 ± 3.38 ^a^	21.52 ± 1.67 ^b^
Tyrosol	10.21 ± 0.04 ^a^	13.20 ± 0.17 ^b^	13.44 ± 0.28 ^b^	28.76 ± 1.11 ^c^
Tyrosol acetate	9.77 ± 0.32 ^a^	4.16 ± 0.66 ^b^	3.26 ± 0.15 ^b^	0.35 ± 0.17 ^c^
p-HPEA-EA	7.02 ± 0.64 ^a^	6.13 ± 0.55 ^a^	5.20 ± 0.38 ^a^	2.54 ± 0.22 ^b^
p-HPEA-EDA	105.49 ± 4.53 ^a^	92.92 ± 2.61 ^a^	83.66 ± 4.18 ^b^	18.49 ± 1.21 ^c^

Note: Values with the same letter within the same row are not significantly different (*p* < 0.05). Experiments were set up in two repetitions and samples for each repetition were analysed in duplicate (*n* = 4).

## Data Availability

The data that support the findings of this study are contained within the article. More information is available from the corresponding author, M.T.M.
